# Measurement, modeling and QALYs

**DOI:** 10.12688/f1000research.25039.1

**Published:** 2020-08-26

**Authors:** Paul C. Langley, Stephen P. McKenna

**Affiliations:** 1 College of Pharmacy, University of Minnesota, Minneapolis, USA; 2 Maimon Research, Tuscon, Arizona, USA; 3 Department of Population Health, University of Manchester, Manchester, UK; 4 Galen Research, Manchester, UK

**Keywords:** Imaginary QALY, ordinal scores, impossible models

## Abstract

Over the past 30 years, a mainstay of health technology assessment has been the creation of modeled incremental cost-per-quality adjusted life year (QALY) claims. These are intended to inform resource allocation decisions. Unfortunately, the reliance on the construction of QALYs from generic utility scales is misplaced. Those advocating QALY-based lifetime modeled claims fail to appreciate the limitations placed on these constructs by the axioms of fundamental measurement. Utility scales, such as those created by the EQ-5D-3L instrument, are nothing more than multidimensional, ordinal scales. Such scales cannot support basic arithmetic operations. Interval scales can support addition and subtraction; ratio scales the further operations of multiplication and division. Those who advocate the construction of QALYs fail to appreciate that such an operation is only possible if the utility scale is unidimensional and has ratio properties with a true zero. The utility measures available do not meet these requirements. As we cannot produce meaningful utility values, the QALY is an invalid construct. Consequently, cost-per-incremental QALY claims are impossible to sustain and the application of cost-per QALY thresholds meaningless. As utility is a latent, unidimensional variable, the best a measure of utility could achieve would be unidimensionality and interval scaling properties. Where such measures are available, they could support claims for response to therapy. Consequently, there would be no need to continue constructing imaginary lifetime value assessment frameworks. Admitting that the QALY is a fatally flawed construct means rejecting 30 years of cost-per-QALY models.

## Introduction

The value framework advocated by the International Society for Pharmacoeconomics and Outcomes Research (ISPOR) is quite clear: *“Leaders in the field of economic evaluation in health care have long recommended that analysts seeking to inform resource allocation decisions approximate the value of interventions in terms of incremental cost per Quality Adjusted Life Year (QALY) gained”* ^1^. The application of this value framework is probably best exemplified in the reference case technology assessment guidelines put in place by groups such as the National Institute for Health and Care Excellence (NICE) in the UK, the Canadian Agency for Drugs and Technologies in Health (CADTH) and the Institute for Clinical and Economic Evaluation (ICER) in the US. In each case pharmaceutical manufacturers and others (including the ICER itself) are asked to make a case for comparative cost effectiveness. This is done by constructing an imaginary (yet apparently believably ‘realistic’) simulation model extending, in the default case, for the lifetime of persons with a chronic disease. The costs and benefits of comparator interventions for the defined hypothetical population are then calculated. Benefits are expressed in terms of incremental cost-per-QALY claims. There is no intention that the resulting claims should meet the standards of normal science for credibility, evaluation and replication ^2^. The model is not about the discovery of new facts; it is purely speculative. This is made clear in the latest version of the Canadian guidelines where it states: *“Economic evaluations are designed to inform decisions. As such they are distinct from conventional research activities, which are designed to test hypotheses”* ^3^. By rejecting the construction of empirically verifiable theories and hypotheses, the imaginary simulated worlds of economic evaluations fail the demarcation test; they are pseudoscience not science ^4^.

## Creating QALYs

There is no ‘gold standard’ measure that can be used to generate QALYs. Several generic multiattribute instruments have been developed for this purpose. These differ considerably and produce markedly dissimilar scores for the same health states. The most used measures are the EQ-5D-3L and EQ-5D-5L, the HUI Mk2 and Mk3 and the SF-6D. These are designed to generate utility or value metrics on a scale from 0 = death to 1 = perfect health. Unfortunately, in the case of the EQ-5D-3L, the most widely used instrument, the algorithms applied to create utility scores can generate negative utility. The same argument, the production of negative utilities, applies to the other instruments. With the EQ-5D-3L utilities are allowed to range from −0.59 to 1.0. The negative utilities generated are considered to indicate states ‘worse than death’. The zero value in each measure is arbitrary, and it is not clear whether a utility of zero or lower makes any sense. The utility value is then applied to the simulated time spent in various hypothetical disease states over the course of a disease and a value adjusted time spent measure created: the QALY. QALYs are then aggregated (and discounted) over the simulated course of the disease to generate lifetime QALYs. Given estimated lifetime costs, the analyst can then produce lifetime cost-per-QALY, and eventually a simulated incremental cost-per-QALY claim.

For the utility value to support these operations it has to meet the axioms of fundamental measurement ^5^. Four main types of measurement scale are recognized: nominal, ordinal, interval and ratio. Each satisfies one or more of the properties of: (i) *identity -* where each value has a unique meaning; (ii) *magnitude* where each value has an ordered relationship to other values; (iii) *interval* where the distances between scale units are equal to one another; and (iv) *ratio* where there is a ‘true zero’ below which no value exists. Nominal scales are purely descriptive and have no inherent numerical value in terms of magnitude. Ordinal scales have both identity and magnitude in an ordered relation but the distance between the ranks can differ considerably, generating only medians and modes (e.g., EQ-5D scales). The interval scale has identity, magnitude and equal intervals. It supports mathematical operations of addition and subtraction. A ratio scale satisfies all properties, supporting the additional operations of multiplication and division.

The question that must be addressed for those supporting QALYs is whether the utility value has ratio measurement properties. If we consider the EQ-5D-3L, there is no evidence that it measures at an interval level, let alone that it has ratio measurement properties. ^5^ Quite the opposite. It can generate negative utilities and then negative QALYs. Put simply, it does not have a true zero. As the EQ-5D-3L is based on symptoms defined by ordinal response levels, the resulting EQ-5D-3L score can only have ordinal properties, not ratio properties. The same argument applies to the other instruments. There is no evidence to suggest that the question of fundamental measurement was considered in its development. The principal objective was a simple, functionally based capture of five symptoms with three ordinal response levels. Across any disease state, patients respond to the same five symptoms. Community preference weights are then applied and an algorithmic value is produced. The result is an ordinal score. Multiplying this score by time spent in a disease state is mathematically impossible.

Unless it can be demonstrated that the EQ-5D-3L (or any other value scale) has ratio properties for any target patient population, the concept of a generic utility QALY collapses; it defies measurement. The implications are interesting: the reference case incremental cost-per-QALY value framework is unintelligible, the claims for simulated QALY based cost-effectiveness claims with willingness to pay thresholds is redundant and some 30 years of advocating the construction of simulated imaginary worlds irrelevant. Rather than seeking real-world evidence, we are locked into a paradigm for imaginary world evidence.

## Abandoning the QALY

Can the QALY be rescued; or, more to the point, do we want to put in the effort to rescue it? Certainly, it could be possible to start from scratch and develop a new measure from first principles employing modern rather than classical test theory measurement. This recognizes the application of Rasch measurement theory (RMT) in its application of conjoint simultaneous measurement (CSM). However, even with the application of RMT, we are unable to develop a scale with ratio properties unless there is a clear specification equation guiding its content ^6^. At best we might develop a value set with interval properties, but this would preclude relating health status to time spent in a disease state (a multiplicative function) to create a QALY.

Do we need a QALY? Is there really a need to talk in terms of incremental cost-per-QALY claims? If we are concerned with quality of life and not the more narrowly defined health-related quality of life that characterizes almost all patient-reported outcome measures (PROMs), then we should consider disease-specific measurements. This is overdue; for we can say unequivocally that PROMs that were developed utilizing classical test theory, will not meet Rasch measurement standards. Quite simply, they were not designed to reflect an underlying latent construct with items selected to conform to Rasch measurement requirements. In some cases, it is possible, *ex post facto*, to ‘rescue’ an instrument through item assessment and possible removal of misfitting items ^7, 8^. A more positive approach would be to go back to first principles, as put forward by Rasch some 60 years ago, and meet fundamental CSM in the development of instruments ^9^.

A further obstacle to rescuing the QALY is the fact that the utility manifest score can take negative values. This has been shown across many disease states for both the EQ-5D-3L and EQ-5D-5L ^10, 11^. In the former, the lowest possible manifest score, as noted above, is −0.59; in the latter the lowest score is −0.29. These negative scores, assuming we ignore the standards of fundamental measurement, lead to the intriguing possibility of negative QALYs. In other words, over a hypothetical lifetime, patients can conceivably hop into and out of negative QALY disease stages. With aggregate lifetime QALYs the sum of the time spent in these positive and negative QALY states could cancel each other out. It is not clear how we would interpret this ordinal score construction of negative time? Particularly where the lifetime summation of QALYs by disease stage is negative: cost per negative QALY?

## Need fulfillment and Rasch

It is a puzzle why those developing PROMs that are focused on functional status and symptom response should ignore the interests of the patient and, often, caregivers. After all, there is no reason why a physician’s view of response to therapy should necessarily be concordant with that of the patient or caregiver. If quality of life has any meaning it should focus on the patient as the principal ‘beneficiary’ of therapy interventions. A patient-centric approach, where life maintains its quality if patient needs are fulfilled, is not a new concept. It was first proposed in the early 1990s and has been the driving force in disease-specific instrument development within the Rasch measurement framework ^12, 13^.

## The Rasch model

Measurement is critical for the advancement of science. The focus, as in the physical sciences, should be on the development of unidimensional indices rather than profiles. We need to focus on one attribute at a time (e.g., temperature ^14^ or pain), not confusing several attributes into a meaningless single score. Despite this, fundamental measurement scales are rare in medicine. If they are to advance beyond ordinal raw scores, they must meet the axioms of invariance and sufficiency ^15^. Where the object to be measured is a latent construct, such as quality of life, we require a framework for identifying, if they exist, inherent measurement structures with interval properties. This is provided in the application of the axioms of conjoint simultaneous measurement developed independently by Rasch, and Luce and Tukey in the early 1960s ^16, 17^. To reflect an underlying unidimensional latent construct such as need-based quality of life, the CSM model argues that two requirements must be met by any outcome measure: (i) item difficulty (the easier the item in a questionnaire, the more likely it is to be affirmed), and (ii) respondent ability (the more able the respondent, the more likely are they to affirm the item).

If we consider quality of life measures, where the latent construct is need fulfillment, the items are generated by qualitative patient interviews in a specific disease state. Where data generated by the measure fit the Rasch model, a single index with interval properties is produced that captures response to therapy. QALYs and imaginary lifetime models are irrelevant. In other words, a patient-centric quality of life measure is generated, not a multi-attribute outcome such as the EQ-5D-3L that confuses a clinically based set of symptoms and responses to produce a meaningless outcome.

This is not to say that the Rasch model has been ignored. There are now several need-based disease-specific quality of life instruments available for clinical trials and for evaluating the impact of competing interventions on quality of life ^18^.

## Next steps

Science can only make significant advances if measures are developed that have the required measurement properties; unidimensionality and ratio level measurement. Utility measures produce composite scores, as they add together several different types of outcome, for example, pain, emotional distress and physical mobility. Composite measurement cannot replace unidimensional measurement.

We have known how to develop unidimensional measures for the last 60 years, through the application of RMT. However, this also requires the development of theoretical models that explain the nature of the outcome that is to be measured and generating relevant content from people who are the true experts (patients in the case of quality of life). Such measurement is rare. Fitting measure data to the Rasch model is also a challenge, because of its strict requirements. For this reason, researchers continue to use dated methodologies and look for measurement models that are less demanding. Unfortunately, the consequences of failing to meet the requirements for fundamental measurement implies that the cost-per-QALY construct is an analytical dead end and much of the utility modeling conducted in the past 30 years has been profitless.

Abandoning the QALY would be, to say the least, embarrassing. A centerpiece of health technology assessment would be shown to have no discernible value. It is not just a question of pointing to the shortcomings of QALYs, but making it clear that the QALY, as exemplified in incremental cost-per-QALY modeled claims, is an impossible construct. Claims for pricing and access for pharmaceutical products and devices must be rejected; they are not realistic.

This article is intended to demonstrate that, in failing to appreciate the axioms of fundamental measurement, the utility values included in QALY analyses are an analytical dead end. If we are to assess the impact on patients of emerging therapies accurately, we need a disease-specific framework that provides a coherent assessment of the comparative benefits to patients and caregivers. We cannot include approximate information as an element in the evidence (real or imaginary) presented to formulary committees. Just as claims based on phase 3 clinical trials are recognized as robust, so should claims for quality of life and utility meet the same standards. This would free us to return to normal science and hypothesis testing.

## Data availability

No data are associated with this article.

## References

[1] Neumann PJ Willke RJ Garrison LP: A Health Economics Approach to US Value Assessment Frameworks – Introduction: An ISPOR Special Task Force Report (1). *Value Health.* 2018;21(2):119–123. 10.1016/j.jval.2017.12.012 29477388

[2] Langley P: Nonsense on Stilts – Part 1: The ICER 2020-2023 value assessment framework for constructing imaginary worlds. *Innov Pharm.* 2020;11(1). 10.24926/iip.v11i1.2444 PMC8132519 34017624

[3] CADTH: Guidelines for the economic evaluation of health technologies. Ottawa.2017 Reference Source

[4] Piglucci M: Nonsense on Stilts: How to tell science from bunk. Chicago; University of Chicago Press.2010 Reference Source

[5] Merbitz C Morris J Grip JC: Ordinal scales and foundations of misinference. *Arch Phys Med Rehabil.* 1989;70(4):308–12. 2535599

[6] Stenner AJ: Measuring reading comprehension with the lexile framework. Paper presented at: Fourth North American Conference on Adolescent/Adult Literacy; Washington, DC.1996 Reference Source

[7] Pallant JF Tennant A: An introduction to the Rasch measurement model: an example using the Hospital Anxiety and Depression Scale (HADS). *Br J Clin Psychol.* 2007;46(Pt 1):1–18. 10.1348/014466506x96931 17472198

[8] Heaney A McKenna SP Hagell P: Improving scoring precision and internal construct validity of the Bath Ankylosing Spondylitis Disease Activity Index (BASDAI) using Rasch Measurement Theory. *J Rheumatol.* 2019;jrheum.180943 10.3899/jrheum.180943 31092712

[9] Bond T Cox C: Applying the Rasch Model. (3 ^rd^Ed.). New York: Routledge.2015 Reference Source

[10] Parkin D Devlin N Feng Y: What determines the shape of an EQ-5D index distribution. *Med Decis Making.* 2016;36(8):941–51. 10.1177/0272989X16645581 27112934

[11] Feng Y Devlin N Bateman A: Distribution of the EQ-5D-5L profiles and values in three patient groups. *Value Health.* 2019;22(3):355–61. 10.1016/j.jval.2018.08.012 30832974

[12] McKenna SP Heaney A Wilburn J: Measurement of patient-reported outcomes. 1: The search for the Holy Grail. *J Med Econ.* 2019;22(6):516–22. 10.1080/13696998.2018.1560303 30556774

[13] McKenna SP Heaney A Wilburn J: Measurement of Patient-Reported Outcomes. 2: Are Current Measures Failing Us? *J Med Econ.* 2019;22(6):523-30. 10.1080/13696998.2018.1560304 30556787

[14] Chang H: Inventing Temperature: Measurement and Scientific Progress. New York: Oxford: University Press.2007 Reference Source

[15] Grimby G Tennant A Tesio L: The Use of Raw Scores From Ordinal Scales: Time to End Malpractice? *J Rehabil Med.* 2012;44(2):97–98. 10.2340/16501977-0938 22334345

[16] Rasch G: Probabilistic models for some intelligence and attainment tests. Copenhagen: Danmarks Paedagogiske. Institut.1960 Reference Source

[17] Luce RD Tukey JW: Simultaneous conjoint measurement. A new type of fundamental measurement. *J Math Psychol.* 1964;1(1):1–27. 10.1016/0022-2496(64)90015-X

[18] Rouse M Twiss J McKenna SP: Co-calibrating Quality-Of-Life Scores From Three Pulmonary Disorders: Implications for Comparative-Effectiveness Research. *J Med Econ.* 2016;19(6):596–603. 10.3111/13696998.2016.1148700 26824603

