# Supplementary material for: Measurement, modeling and QALYs
Source: F1000Res. 2020 Aug 26;9:1048. [Version 1] doi: 10.12688/f1000research.25039.1 (PMC7533730; doi:10.12688/f1000research.25039.1)
Supplement: Peer review history

# Peer review: Reviewer response for version 1

**DOI:** 10.5256/f1000research.27623.r72004

**Published:** 2020-10-02

**Authors:** Jonathan Belsey

**Affiliations:** 1 JB Medical Ltd, Sudbury, UK

This paper considers the mathematical underpinnings of current methods used to quantify utility and considers the implications the shortcomings that he highlights may have for the interpretation of QALYs and consequently the results of traditional cost utility analyses.

 The authors highlight the fact that, in order to support the manipulations inherent to a cost utility model, the tool used to generate utility must satisfy a number of requirements (unidimensionality, ratio properties, true zero), without which it is not meaningful to carry out the arithmetic manipulations inherent in a cost utility approach.

 Using the EQ-5D-3L instrument as an example, the authors demonstrate that these requirements are signally not met, and in consequence, the conclusions of analyses based on this approach are fatally undermined.

 I cannot fault the authors' chain of logic and in consequence, must agree with their conclusions. This is not a qualitative surprise - anyone who works in health economics is well aware that the QALY is a flawed measure - but I had never really thought through the mathematical inconsistencies before, in the detailed and logical way that the authors highlight.

 One would like to think that we could behave as scientists and begin to look at how we can modify our approach to the economic assessment of healthcare interventions to address these shortcomings. Unfortunately, it seems likely that there is too much political and intellectual capital invested in the QALY to anticipate its downfall any time soon. Unfortunately, there is a long and undistinguished history of misused metrics remaining embalmed in our approach to data analysis on the basis that: "It may be flawed, but at least it is equally flawed for everyone". I fear that the QALY will continue to dictate healthcare spending for a good many years yet, but I nonetheless congratulate the authors for their incisive critique of its derivation.

Is the rationale for developing the new method (or application) clearly explained?

Yes

Is the description of the method technically sound?

Yes

Are the conclusions about the method and its performance adequately supported by the findings presented in the article?

Yes

If any results are presented, are all the source data underlying the results available to ensure full reproducibility?

No source data required

Are sufficient details provided to allow replication of the method development and its use by others?

Yes

Reviewer Expertise:

Health economics and biostatistics

I confirm that I have read this submission and believe that I have an appropriate level of expertise to confirm that it is of an acceptable scientific standard.

# Peer review: Reviewer response for version 1

**DOI:** 10.5256/f1000research.27623.r70294

**Published:** 2020-09-07

**Authors:** Ariel Beresniak

**Affiliations:** 1 Data Mining International, Geneva, Switzerland

The article (Measurement, modeling and QALYs) is a methodological review paper about the limitations of the QALY indicator used in health decision making.

 Compared to the high number of publications presenting economic models in many therapeutic areas expressed in incremental costs per QALY, few papers have been published explaining the criticisms of QALY calculation, confirming the interest of this paper in the scientific littérature.

 This article presents clearly the basic metric properties necessary to allow calculations and why the QALY indicator does not fulfill the conditions to be used in economic assessments.

 Some explanations are missing for such methodological review. Firstly the multiplicative formula should be presented for calculating QALY. Secondly, the assumptions underlying the QALY approach should be discussed to better understand the controversy.

 A reference to the ECHOUTCOME European project specifically testing the validity of the QALY assumptions and concluding that the QALY approach is flawed should be mentioned as the most extensive research about the dangers of using QALY in health decision making, and how this new paper confirms or complete the findings. ^1^

Is the rationale for developing the new method (or application) clearly explained?

Yes

Is the description of the method technically sound?

Partly

Are the conclusions about the method and its performance adequately supported by the findings presented in the article?

Yes

If any results are presented, are all the source data underlying the results available to ensure full reproducibility?

No source data required

Are sufficient details provided to allow replication of the method development and its use by others?

Yes

Reviewer Expertise:

Methodology in health decision making

I confirm that I have read this submission and believe that I have an appropriate level of expertise to confirm that it is of an acceptable scientific standard.

## References

[1] : Validation of the underlying assumptions of the quality-adjusted life-years outcome: results from the ECHOUTCOME European project. *Pharmacoeconomics*.2015;33(1) : 10.1007/s40273-014-0216-0 61-9 10.1007/s40273-014-0216-0 25230587
